# Factors associated with the health and nutritional status of children under 5 years of age in Afghanistan: family behaviour related to women and past experience of war-related hardships

**DOI:** 10.1186/1471-2458-8-301

**Published:** 2008-08-29

**Authors:** Taufiq Mashal, Takehito Takano, Keiko Nakamura, Masashi Kizuki, Shafiqullah Hemat, Masafumi Watanabe, Kaoruko Seino

**Affiliations:** 1 Health Promotion Section, Division of Public Health, Graduate School of Tokyo Medical and Dental University, Tokyo, Japan; 2 General Directorate of Preventive Medicine and Primary Health Care, Ministry of Public Health, Kabul, Afghanistan; 3 International Health Section, Division of Public Health, Graduate School of Tokyo Medical and Dental University, Tokyo, Japan

## Abstract

**Background:**

The present study was performed to assess, beyond socio-economic factors, independent associations between the health and nutritional status of children under 5 years old and (1) family behavioural factors related to women with regard to child care and (2) war-related experience by the household of hardships in Afghanistan.

**Methods:**

The subjects were all children born during the previous 5 years from 1400 households in Kabul Province, Afghanistan and were selected by multistage sampling in March 2006. Height and weight measurements of the children and culturally sensitive interviews with their mothers were conducted by household visits. Child mortality, morbidity and nutritional status were evaluated. Four areas were assessed as variables for family behavioural factors related to women: education of mothers, child marriage of the mothers, maternal autonomy in obtaining healthcare for children and preference for a female physician. Hardships experienced by the family were examined by determining their satisfaction of basic material needs and by any experience of being forced to leave a preferred residence.

**Results:**

A total of 2474 children from 1327 households completed the examinations and interviews; among them, 101 children were deceased by the time of the interview visits. Diarrhoea (32.5%) and acute respiratory infection (41.0%) were common child health problems and both emaciation (12.4%) and linear growth retardation (39.9%) were prevalent. Regardless of the influence of economic, demographic, family behavioural or hardships experience factors, a lack of maternal autonomy (79.1%) was associated with the occurrence of acute respiratory infection (odds-ratio = 1.72; 95% confidence interval = 1.23, 2.40), and linear growth retardation of children (odds-ratio = 1.38; 95% confidence interval = 1.01, 1.90); a lack of education of the mother (71.7%) and child marriage of the mothers (18.3%) were associated with diarrhoea (odds-ratio = 1.84; 95% confidence interval = 1.40, 2.41; odds-ratio = 1.46; 95% confidence interval = 1.08, 1.96, respectively); a shortage of basic material needs (59.1%) was associated with diarrhoea (odds-ratio = 1.35; 95% confidence interval = 1.08, 1.68); and migration inside the country (52.9%) was associated with underweight children (odds-ratio = 2.48; 95% confidence interval = 1.13, 5.44).

**Conclusion:**

A lack of education of the mothers, child marriage, lack of maternal autonomy, shortage of basic material needs and internal displacement showed independent and significant negative associations with child health and nutritional variables in this country that has experienced a long period of conflict.

## Background

Ten and half million children under 5 years of age die every year worldwide, with 98% of these deaths occurring in developing countries [1]. Diarrhoea and respiratory diseases are major causes of child mortality, and the high prevalence rates of emaciation and linear growth retardation reflect the poor health status of children in developing countries [1,2]. The health indicators of Afghanistan, a country located in South Asia with a population of 23 million, indicate one of the worst child mortality rates in the world [3,4]. Although some progress in child health has been seen since 2003 after a long-period of armed conflict in the country, child survival in Afghanistan continues to be an issue of national and international concern. A recent survey indicated that the mortality rate for children under 5 years in Afghanistan is 191 out of 1000 live births [1,5]. The country's economy and development are fragile with a gross domestic product per capita of US$290 and a human development index of 0.312, ranked 169 among 177 countries in the world [6]. The people of Afghanistan experienced a long period of armed conflict from 1979 to 2001. The Office of the United Nations High Commissioner for Refugees estimates that 3.5 million refugees have returned to their homes since the end of the war [7,8].

Previous studies have indicated close correlations between the health of children in developing countries and a number of individual determinants of child health, such as family income [9,10], the education of mothers [11,12], sanitation [13,14] and immunisations[15,16]. Other studies addressed comprehensive factors associated with health within the populations [17,18]. To identify the actual priorities within a society, it is necessary to determine not only the associations between individual variables and child health, but also the comprehensive associations of health-related factors in a population.

While some of the above factors associated with health are widely recognised, a range of social factors in diverse sociocultural environments may also be related to health. Various behavioural patterns are closely linked to the values of families. In some cultures, social norms within the community restrict patterns of women's behaviour [19,20]. Among these behaviours, maternal autonomy under the influence of family behaviour is expected to be critical in securing better healthcare for children, as mothers are the primary caregivers for their children. Discussion and measurement of women's autonomy can be extended to study the relationship between maternal autonomy and common child health problems. Although common since ancient times, there are serious concerns regarding child marriage. A recent study indicated that 48% of women aged 15 to 24 years in South Asia were married before they were 18 years old [21].

Armed conflict places health security at risk as it makes it difficult to obtain food, water and shelter, thus disrupting the basic material requirements for a healthy life [22]. Families and young children are at increased risk of becoming victims of a lack of basic material needs and stressful living conditions [23]. Freedom to choose a place of residence has been repeatedly jeopardised in Afghanistan during the armed conflicts, and an estimated one third of the population were either internally or externally displaced during these conflicts [8,24]. The human security needs are referred to as satisfaction of basic material needs (food, water and adequate shelter), schooling for children, protection from violence and a state that does not oppress its citizens but governs with their consent [22]. There is concern regarding the consequences on child health of a lack of basic material needs, but to date there have been no discussions of this issue based on accumulated evidence. Afghanistan has been classified as 'post-conflict' since the end of 2001, but the security situation remains fragile in many parts of the country [25]. Therefore, the influence of conflict on the health of Afghan children is a critical concern.

Since 2002, there has been an increase in efforts to improve the health of children through the elimination of poverty, expansion of basic health services and education for girls [26,27]. To optimise these efforts and elucidate the comprehensive factors required for improvement of the health of children in a country under post-conflict conditions, it is necessary to obtain not only estimated information representing the whole country or information from patients that are able to reach hospitals [28,29], but also evidence from those actually living in the affected communities.

Socio-economic and environmental factors at the household level, as well as the characteristics of mothers, have been shown to be associated with both the health and nutritional status of children less than 5 years old in most developing countries. The present study was performed to assess, beyond these socio-economic factors, independent associations between the health and nutritional status among children under 5 years of age and (1) family behavioural factors related to women with regard to child care and (2) war-related experience by the household of hardships in Afghanistan.

## Methods

### Subjects

The study was performed in Kabul Province, an area with a total population of 3.1 million in 2006. The target and source population consisted of all children born after 20 March 2001, the date of the Afghan New Year, in Kabul Province (that is, those under 5 years of age at the time of the survey: n = 614,320). The survey was household-based, with a household defined as a group of people who usually take their meals together from the same cooking pot, share household assets and accumulate their earnings to procure household materials.

The sampling of households was conducted by multistage sampling. First, two districts were selected from Kabul Province: one selected from among seven urban districts and the other selected from among seven rural districts. A total of 700 households in three out of eight sub-districts in the urban district and 700 households in three out of seven sub-districts in the rural district were selected by trained health workers in the same geographic areas following the sampling procedures. Supervisors and coordinators of the Expanded Programme on Immunisation familiar with the area and the households with children cooperated with health workers to select the households. The criteria for inclusion of a subject household in the study was that it had had newborn children in the previous 5 years, regardless of their survival status (alive/deceased) at the time of the household sampling. Households that had lived in the area during the previous 5 years or those that had lived in other places were included as subject households. Households were chosen by survey teams in each sub-district. A household randomly selected from a map of the sub-district was contacted and 10 households satisfying the study criteria were selected. The selections were repeated until the number of households reached the number allocated for the individual sub-districts. These lists of eligible households in the selected primary units of districts were developed between 11 and 18 March 2006.

### Survey procedures

The survey teams performed health examinations of children and face-to-face interviews with their mothers or female caregivers using a structured questionnaire. Ten teams were assigned to visit the subject households between 20 March and 22 April 2006.

Each team consisted of one female interviewer and one male recorder trained in household health surveys taking into consideration the cultural sensitivity of Afghan women who generally refrain from communicating with males outside their immediate families. According to local customs, when required, an additional close legal male relative (*Mahram*) of the female interviewer was assigned to accompany individual survey teams to facilitate mobility of the female interviewer in the community.

Interviewers and recorders had been well trained and were conversant with both the survey procedures and questionnaire study. The quality of examinations and interviews by individual survey teams were standardised by repeated pre-tests. Completeness and consistency of recording on the questionnaire sheets were evaluated by survey supervisors at the end of each working day, and any omissions or inconsistent reporting were re-examined by revisits to the relevant household. Data entry was performed by three investigators independently and triplicate databases were cross-checked using Microsoft Office Excel version 2003.

The study protocol was approved by the Ethical Review Board Committee of the Ministry of Public Health Afghanistan (358308) as of 26 December 2005. In addition, permission was obtained from the heads of district governments and community/religious leaders.

### Measurements

#### Health of children

The survival status, history of major illness and nutritional growth of the subjects were evaluated.

Deaths of the subjects from any causes at any dates up to the interview were examined. The recent encouragement in recording children's date of birth in accordance with the political stability in the country provided reliable demographic information on which to base results.

Assessments of major illnesses were made through culturally sensitive interviews with the mothers using a structured questionnaire. Major illnesses were defined according to the following two conditions: acute diarrhoea as the passage of watery/soft stools three or more times in 24 hours during the 2 weeks prior to the interview, and an episode of acute respiratory infection (ARI) was defined as having a cough/cold or difficulties in breathing during the last 2 weeks prior to the interviews [29].

Anthropometric examinations of the surviving subjects were performed to evaluate nutritional growth of children, in accordance with the World Health Organization (WHO) guidelines for field assessment [30]. The age of children in months and years was assessed according to interviews with the mothers by considering the calendar year or well-recognised historical events in Afghanistan. Emaciation, linear growth retardation and under-weight were defined as weight-for-height, height-for-age and weight-for-age less than -2 *z*-scores according to the WHO 2006 growth reference [31], respectively. Outlying values of *z*-scores were defined as lower than -6 or greater than 5 for weight-for-age, lower than -6 or greater than 6 for height-for-age, and lower than -5 or greater than 5 for weight-for-height and not used in the analysis, according to the WHO manual [31]. A macro for SPSS, which included the WHO 2006 growth standard to calculate nutritional indicators, was used for the calculation [32]. We also calculated the indicators of emaciation, linear growth retardation and under-weight represented by *z*-scores of weight-for-height, height-for-age and weight-for-age using the WHO-National Center for Health Statistics (NCHS) reference with Epi Info version 3.4.3 (Centers for Disease Control and Prevention, Atlanta, Georgia, US) for comparative purposes.

#### Economic and environmental factors

In the context of Afghanistan, women have the main responsibility within the household for safeguarding the husband's income and using it for daily expenses. Agriculture plays a relatively small role in supporting the household economy in Kabul and household incomes from formal and informal sources were assessed. In families not headed by a man earning a regular salary, efforts were made to obtain the best estimate in this category. Based on the terciles, families were divided into three categories according to monthly household income: low (38.8%), middle (26.5%) and high (34.7%).

Household sanitary conditions were evaluated based on the availability of running water. Missing the third dose of diphtheria, pertusis and tetanus (DPT3) vaccine (after exclusion of children aged 3 months or less) was considered an indicator of a lack of utilisation of preventive healthcare services.

#### Family behavioural factors related to women with regard to child care

Behaviours of families in Afghanistan relate closely to the behaviour of women with regard to child care. The following conditions were included in the analysis: mothers who had not attended school for at least a year were regarded as having a lack of school education; child marriage of mothers was defined by their first delivery under the age of 16 years; maternal autonomy in seeking healthcare at the household level was based on the grouping of the two variables of 'requirement for permission from the head of the household to bring the child to see a doctor' and 'requirement for an accompanying person to visit a health facility with the child'; and mother's preference for a physician of a specific gender to seek consultation for the child. The reasons for the mother's preference for a physician of a specific gender were also examined. Information was obtained on all of these variables by direct answers from the mothers.

#### Experience of hardship

Experiences of a shortage of basic material needs and residence displacement were examined. The variable 'shortage of basic material needs' was constructed from initial variables of shortage of food, water and shelter. Questions regarding each of these variables had answers of 'no', 'less than 1 year' and '1 year or more'. First, we combined the 'no' and 'less than 1 year' period and coded them '0' with a period of '1 year or more' coded as '1'. In the second stage, we combined the codes of the three variables for shortage of food, water and shelter, and created a single proxy variable for a shortage of basic material needs. Periods of 1 year or longer of shortages of food, water or shelter and experiences of forced displacement, with or without migration outside the country, were identified. In the majority of cases, migration outside the country after forced displacement was defined as refugee status, while experience of forced displacement without migration outside the country was defined as internal displacement.

### Statistical analysis

Mortality (defined as the number of deaths among the subjects), the prevalence of major illnesses among surviving children and child growth indicators were calculated and used as child health indicators. Family behavioural factors related to women with regard to child care and experiences of hardship were regarded as potential factors affecting child health. We conducted a statistical analysis of the data with ordinal procedure because the 'design effect' on the study subjects sampled from 140 units is considered to be small.

Data of both living and deceased subjects were used for analyses of mortality, while data on deceased subjects were excluded from analyses on the prevalence of morbidity of major illness and child nutritional status.

The associations among child health indicators and potential factors affecting the health of children were first examined by an adjusted logistic regression analysis.

Multivariate logistic regression models were applied to evaluate independent associations among child health indicators as dependent variables and one of the factors of family behaviour or experience of hardships. Odds-ratios (ORs) were calculated by adjusting the influence of household economics, age and sex of children, mother's age, availability of running water and other variables of family behavioural factors and household experience of hardship (that is, education of the mother, child marriage, maternal autonomy, preference for female physician, shortage of basic material needs, forced to leave preferred residence, migration inside the country, migration outside the country). For all variables of family behaviour except the mother's preference for consulting a physician of a specific gender and all variables of experience of hardships, a response of 'no' was used as a reference category. For the variable of 'mother's preference for consulting a physician of a specific gender regarding the health of her children', a response of 'either gender' or 'female' was used as a reference category.

Statistical analyses were performed using SPSS for Windows (Rel. 13.0, 1 September 2004; SPSS Inc., Chicago, IL, US). Subjects with missing data for at least one of the explanatory or adjustment variables were individually excluded from the multivariate analysis model. An alpha-type error was regarded as 0.05.

## Results

A total of 1327 households (94.8%) participated in the study. The total number of children born in the previous 5 years among these households was 2474, and consisted of 1280 boys (51.7%) and 1194 girls (48.3%). Interviews were conducted with 1293 mothers and 34 female caregivers (referred to hereafter as 'mothers').

Among the 2474 children included in the analysis who were born in the preceding 59 months, 101 (4.1%) were deceased. The highest numbers of deaths (24) were reported from the children born in the preceding 0–11 months, and the number of deaths by the time of birth was 22, 21, 21 and 13 for children born at 12–23, 24–35, 36–47 and 48–59 months, respectively. The percentages of deceased children reported in the previous 5 years among the subject boys and girls were 4.4% and 3.8%, respectively.

The mean and standard deviation (SD) for children and mothers' age were 2.80 (1.38) and 21.7 (3.6) years, respectively. The means for height and weight of the children were 81.5 cm and 11.7 kg with SDs of 16.8 and 6.2, respectively.

Table 1 shows family behavioural factors related to women, household experience of hardships, household economic status, characteristics of the child and access to health services among the 2373 surviving children. Around 71.7% of the mothers studied had not attended school for more than 1 year. First delivery before the age of 16 was reported in 18.3% of the mothers in the study population. This indicated a prevalent lack of education of mothers. The high percentage of mothers requiring permission from the head of the households or requiring a person to accompany them to visit a healthcare provider (79.1%) indicated that lack of maternal autonomy was prevalent in this area. A total of 772 mothers (58.4%) expressed a preference for consulting with only female physicians regarding the health of their children. The major reasons for this preference were *Mahram *(39.8%), ease in talking to the physician (38.7%) and confidentiality (11.8%). Experience of shortages of food, water or shelter for longer than 1 year was reported in 59.1% of cases. Of the 1119 households reporting having been forced to leave their preferred residence (85.0%), 696 (52.9% of all subjects) reported displacement within Afghanistan, while the rest reported living outside Afghanistan either by their own decision or the decisions of others.

**Table 1:** Family behavioural factors, age of mother, household experience of hardships, household economic status, characteristics of the child and access to health services (*n *= 2373 surviving children in *n *= 1327 households)

**Family behavioural factors related to women**	***n***	**%**
Lack of education (y vs n)	1327	71.7
Married younger than 16 years (y vs n)	1308	18.3
Lack of maternal autonomy (y vs n)	303	79.1
Preference for female physician (y vs n)	1322	58.4
		
**Age of the mother**		

Age of the mother (years)	1322	
13 to 20		38.3
21 to 22		26.8
23 to 38		34.9
		
**Household experience of hardships in the last 5 years**		

Shortage of basic material needs	1227	59.1
Forced to leave preferred residence	1316	85.0
Migration inside the country	316	52.9
Migration outside the country	316	32.1
		
**Household economic status**		

Monthly income terciles (Afghani)	1322	
First (100 to 4999)		37.9
Second (5000 to 9999)		25.6
Third (10,000 to 60,000)		36.5
No running water	1327	38.1
		
**Characteristics of the child and access to health services**		

Female	2373	48.4
Age (months)	2373	
0–11		22.3
1 December 2003		24.3
24–35		19.7
36–47		17.7
48–59		15.9
Missing diphtheria, pertusis and tetanus vaccination (y vs n)	2304	17.9

Table 2 shows the prevalence of morbidity and nutritional status by gender and age among 2373 surviving children. Around 32.5% and 41.0% had suffered from diarrhoea and ARI respectively, in the preceding 2 weeks,

**Table 2:** Prevalence of morbidity and nutritional status by gender and age of the child (*n *= 2373 surviving children)

	**Morbidity**						**Nutritional status**							
			
	**Diarrhoea in the last 2 weeks**			**Acute respiratory infection in the last 2 weeks**			**Emaciation (weight-for-height less than -2*z*)**			**Linear growth retardation (height-for-age less than -2*z*)**			**Under-weight (weight-for-age less than -2z)**	
	***n***	**%**		***n***	**%**		***n*^1^**	**%**		***n*^1^**	**%**		**n^1^**	**%**
**Girls**														

0–11 months	246	31.7		227	47.6		188	18.1		207	40.6		238	39.1
12–23 months	277	43.3		263	36.5		245	10.6		246	37.4		271	13.7
24–35 months	226	31.4		211	44.1		210	11.9		212	43.4		226	28.3
36–47 months	220	27.7		185	43.2		205	8.3		206	41.3		220	21.8
48–59 months	172	18.6		152	37.5		148	11.5		149	28.2		157	17.8
														
All	1141	31.7		1038	41.8		996	11.9		1020	38.7		1112	24.3
**Boys**														

0–11 months	281	36.7		262	43.5		221	23.5		219	37.4		265	37.0
12–23 months	297	38.7		281	43.1		270	13.7		267	41.6		291	20.3
24–35 months	240	37.1		219	32.9		213	10.3		212	43.4		234	23.9
36–47 months	199	28.1		182	40.7		178	6.2		179	40.8		195	21.0
48–59 months	203	21.2		174	39.7		156	7.7		162	42.0		176	15.3
														
All	1220	33.3		1118	40.3		1038	12.9		1039	41.0		1161	24.2
														

All	2361	32.5		2156	41.0		2034	12.4		2059	39.9		2273	24.2

^1^Children with missing values for anthropometric variables or outlying values for nutritional indices are excluded.

The means and SDs of *z*-scores of weight-for-height, height-for-age and weight-for-age were -0.99 (6.81), -1.00 (2.71) and -0.57 (2.08), respectively, indicating general under-development compared with standard growth charts. These results excluded the 183, 253 or 48 subjects who showed outlying values of weight-for-height, height-for-age and weight-for-age, respectively, according to the WHO 2006 procedure [31]. Emaciation, linear growth retardation and under-weight were observed among 12.4%, 39.9 and 24.2% of the subjects, respectively, according to WHO 2006 [31]. The prevalence of these under-developments calculated using the WHO-NCHS reference were 13.6%, 33.1% and 25.8%, respectively. Percentages of emaciation (wasting) and under-weight by using two definitions were similar, while the percentage of linear growth retardation (stunting) was higher than that calculated by the WHO-NCHS reference. Similar relationships between the results obtained by WHO 2006 and WHO-NCHS references were reported from another study [33].

Table 3 shows the unadjusted associations among child health indicators and family behavioural factors, household experience of hardships, household economic status, characteristics of the child and access to health services. A lack of maternal education, child marriage, lack of maternal autonomy, mothers' specific preference for female physicians, a lack of basic material needs, internal displacement and low economic status all showed significant associations with poor child health. Older children were more likely to suffer from diarrhoea, but less likely to be emaciated or under-weight. The gender of the children did not show any significant associations with the health indicators examined.

**Table 3:** Unadjusted associations of child health indicators with family behavioural factors, household past experience of hardships, household and children characteristics

	**Diarrhoea in the last** **2 weeks**				**Acute respiratory** **infection in the last** **2 weeks**				**Emaciation** **(weight-for-height less than** **-2*z*)**				**Linear growth retardation** **(height-for-age less than** **-2*z*)**				**Under-weight** **(weight-for-ageless than** **-2*z*)**			
	***n***	**OR**	**CI**		***n***	**OR**	**CI**		***n***	**OR**	**CI**		***n***	**OR**	**CI**		**n**	**OR**	**CI**	

**Family behavioural factors related to women**																				
Lack of education (y vs n)	2361	2.28	1.85	2.83	2154	1.00	0.83	1.21	2034	1.33	0.97	1.80	2059	1.55	1.26	1.89	2273	1.62	1.29	2.04
Married younger than 16 years (y vs n)	2325	1.64	1.33	2.03	2121	1.18	0.95	1.46	2001	1.18	0.85	1.63	2029	0.97	0.77	1.21	2237	1.41	1.12	1.78
Lack of maternal autonomy (y vs n)	2316	1.91	1.44	2.53	2112	1.65	1.25	2.18	1995	1.45	0.95	2.21	2021	1.77	1.34	2.32	2229	1.88	1.36	2.61
Preference for female physician (female vs either)	2349	1.41	1.18	1.68	2144	1.19	1.01	1.42	2023	1.07	0.82	1.40	2049	1.37	1.14	1.64	2261	1.39	1.14	1.70
**Household experience of hardships**																				

Shortage of basic material needs (y vs n)	2182	1.65	1.37	1.98	1983	1.15	0.96	1.37	1888	0.83	0.63	1.09	1904	1.28	1.06	1.54	2104	1.24	1.01	1.53
Forced to leave preferred residence (y vs n)	2338	1.39	1.08	1.80	2133	0.84	0.67	1.07	2019	1.24	0.83	1.86	2041	1.39	1.07	1.81	2251	1.42	1.06	1.90
Migration inside the country (y vs n)	2338	1.44	1.21	1.72	2133	0.92	0.77	1.09	2019	1.32	1.00	1.73	2041	1.27	1.06	1.52	2251	0.68	0.55	0.85
Migration outside the country (y vs n)	2252	0.78	0.64	0.94	2047	0.95	0.79	1.14	1942	0.76	0.56	1.02	1965	0.88	0.73	1.06	2168	1.67	1.37	2.04
**Household economic status**																				

Income (vs high)	820				743				727				742				803			
Low	910	1.68	1.37	2.07	832	1.20	0.97	1.48	770	1.25	0.92	1.69	769	1.20	0.98	1.48	860	1.51	1.21	1.90
Middle	618	1.37	1.09	1.72	568	1.39	1.11	1.75	526	0.99	0.69	1.40	536	1.09	0.87	1.37	597	1.02	0.79	1.32
No running water	2297	1.51	1.26	1.80	2095	1.08	0.91	1.29	1982	1.14	0.87	1.53	2005	1.61	1.33	1.94	2209	1.48	1.21	1.83
**Characteristics of the child and access to health services**																				

Gender (girl vs boy)	2361	1.07	0.90	1.28	2156	0.94	0.79	1.12	2034	0.92	0.70	1.19	2059	0.91	0.76	1.08	2273	1.00	0.83	1.22
Age (vs 0–11 months)	527				489				409				426				503			
12–23 months	574	1.33	1.04	1.69	544	0.92	0.72	1.19	515	0.52	0.37	0.75	513	1.03	0.79	1.33	562	0.34	0.25	0.45
24–35 months	466	1.00	0.77	1.30	430	1.02	0.78	1.34	423	0.47	0.32	0.69	424	1.20	0.91	1.58	460	0.58	0.44	0.76
36–47 months	419	0.74	0.56	0.98	367	0.89	0.67	1.19	383	0.30	0.19	0.47	385	1.09	0.82	1.44	415	0.45	0.33	0.60
48–59 months	375	0.48	0.35	0.65	326	0.93	0.69	1.24	304	0.40	0.25	0.62	311	0.86	0.63	1.16	333	0.32	0.23	0.45
Missing diphtheria, pertusis and tetanus vaccination (y vs n)	2197	1.67	1.33	2.11	2014	1.57	1.24	1.99	1941	1.34	0.95	1.90	1959	1.48	1.16	1.88	2154	1.98	1.55	2.54

OR = odds-ratios; CI = confidence interval.

Table 4 shows the adjusted associations between child health indicators, family behavioural factors and household experience of hardships calculated by multiple logistic regression analysis. A lack of maternal autonomy was associated with the occurrence of acute respiratory infection (OR = 1.72; 95% confidence interval (CI) = 1.23, 2.40); linear growth retardation (OR = 1.38; 95% CI = 1.01, 1.90); child marriage of the mothers was associated with the occurrence of diarrhoea (OR = 1.46; 95% CI = 1.08; 1.96); shortage of basic material needs was associated with diarrhoea (OR = 1.35; 95% CI = 1.08, 1.68); and migration inside the country was associated with the occurrence of under-weight children (OR = 2.48; 95% CI = 1.13, 5.44).

**Table 4:** Adjusted associations of child health indicators with family behavioral factors and household past experience of hardships

	**Morbidity**																	
							
	**Diarrhoea in the last 2 weeks** **(n = 1,926)**						**ARI in the last 2 weeks** **(n = 1,733)**											
								
	**Model 1**			**Model 2**			**Model 1**			**Model 2**								
**Family behavioral factors related to women**	**OR**	**C.I**.		**OR**	**C.I**.		**OR**	**C.I**.		**OR**	**C.I**.							
							
*Lack of education (y. vs. n.)*	2.10	1.69	2.61	1.84	1.40	2.41	1.01	0.83	1.23	0.85	0.66	1.11						
*Married < 16 y (y. vs. n.)*	1.56	1.26	1.93	1.46	1.08	1.96	1.21	0.97	1.51	1.24	0.91	1.69						
*Lack of maternal autonomy (y. vs. n.)*	1.75	1.32	2.34	1.28	0.92	1.78	1.62	1.22	2.14	1.72	1.23	2.40						
*Preference for female physician (female. vs. either.)*	1.32	1.10	1.59	1.03	0.83	1.29	1.18	0.99	1.41	0.84	0.67	1.06						
																		
**Household experience of hardships**																		
*Shortage of basic material needs (y. vs. n.)*	1.60	1.32	1.93	1.35	1.08	1.68	1.18	0.99	1.42	1.23	0.99	1.54						
*Forced to leave preferred residence (y. vs. n.)*	1.30	1.01	1.69	1.52	0.79	2.94	1.20	0.94	1.52	1.21	0.63	2.33						
*Migration inside the country (y. vs. n.)*	1.40	1.17	1.67	1.60	0.77	3.32	1.12	0.94	1.34	1.57	0.76	3.25						
*Migration outside the country (y. vs. n.)*	0.80	0.66	0.97	0.66	0.33	1.32	1.02	0.85	1.23	0.78	0.39	1.55						

	**Nutritional status**																	
	
	**Emaciation (weight for height < -2z)** **(n = 1,673)**						**Linear growth retardation** **(height for age < -2z) (n = 1,688)**						**Underweight (weight for age < -2z)** **(n = 1,851)**					
		
	**Model 1**			**Model 2**			**Model 1**			**Model 2**			**Model 1**			**Model 2**		

**Family behavioral factors related to women**	**OR**	**C.I**.		**OR**	**C.I**.		**OR**	**C.I**.		**OR**	**C.I**.		**OR**	**C.I**.		**OR**	**C.I**.	
	
*Lack of education (y. vs. n.)*	0.81	0.59	1.11	1.03	0.69	1.53	1.53	1.24	1.88	1.22	0.95	1.57	1.49	1.18	1.88	1.10	0.82	1.48
*Married < 16 y (y. vs. n.)*	1.12	0.80	1.56	1.14	0.71	1.82	0.94	0.75	1.18	0.73	0.53	1.00	1.33	1.05	1.68	1.25	0.90	1.75
*Lack of maternal autonomy (y. vs. n.)*	1.36	0.89	2.09	1.67	1.00	2.81	1.74	1.32	2.29	1.38	1.01	1.90	1.74	1.26	2.42	1.46	1.00	2.14
*Preference for female physician (female. vs. either.)*	1.03	0.78	1.35	0.94	0.67	1.31	1.34	1.11	1.61	1.02	0.82	1.28	1.33	1.08	1.63	0.99	0.77	1.27
																		
**Household experience of hardships**																		
*Shortage of basic material needs (y. vs. n.)*	0.81	0.61	1.07	0.71	0.51	0.98	1.26	1.04	1.52	1.07	0.87	1.33	1.21	0.98	1.49	1.02	0.80	1.31
*Forced to leave preferred residence (y. vs. n.)*	1.19	0.79	1.79	0.61	0.18	2.07	1.04	1.35	1.76	1.08	0.57	2.05	1.36	1.01	1.82	1.67	0.83	3.35
*Migration inside the country (y. vs. n.)*	1.28	0.97	1.68	1.14	0.30	4.32	1.25	1.04	1.49	1.23	0.60	2.53	1.61	1.32	1.97	2.48	1.13	5.44
*Migration outside the country (y. vs. n.)*	0.78	0.58	1.05	1.25	0.35	4.51	0.89	0.74	1.08	0.85	0.43	1.68	0.71	0.57	0.88	0.59	0.28	1.24

Model 1: odds-ratios are adjusted for household economic status, age and sex of children, and age of mother.

Model 2: odds-ratios are adjusted for household economic status, age and sex of children, age of mother, no running water and other variables of family behavioural factors and household experience of hardship (education of mother, child marriage, maternal autonomy, preference for female physician, shortage of basic material needs, forced to leave preferred residence, migration to inside of the country, migration to outside of the country).

## Discussion

Based on general household sampling from communities in Kabul Province, the results of the present study revealed factors associated with the health of children in Afghanistan. Our study revealed a difficult overall context of child health in Afghanistan stemming from easily preventable causes. We found that 32.5% and 41.5% of children suffered from acute diarrhoea and ARI, respectively. The prevalence of emaciation and linear growth retardation was 12.4% and 39.9%, respectively. A lack of maternal autonomy in obtaining healthcare for their children was associated with the prevalence of diarrhoea and poor growth of children. Experience of internal displacement and shortage of basic material needs were negatively associated with child health.

The results of this study represent the first evidence from a cross-sectional study with a community-representative sample regarding comprehensive factors associated with the health of children in a country experiencing conflicts. The high response rate, 94.8%, was regarded as a result of the consideration given to gender sensitivity and other locally relevant factors. Good communication with the authorities throughout the research process aided in both security in the study area and in the collection of quality data. We used recent political changes in the country as milestones to minimise the effect of lack of respondent's precision regarding the accuracy of their children ages. Careful questioning by considering local relevance and by giving a practical definition to the reporting was regarded as minimising misclassification.

This study had a cross-sectional design and therefore, direct causality could not be determined. Although recall bias is possible when collecting information regarding retrospective events, we attempted to minimise the effect by limiting retrospective reporting of child illness to the 2 weeks prior to the interview and this was regarded as minimising under-reporting or misclassification. A larger number of deaths was reported from children born in the previous 12 months compared with those born at 48 months or longer. This might be explained by the non-reporting of birth of children already deceased. To facilitate further understanding of mortality by age, a prospective research study with complete follow-up is required.

The child mortality rate defined in this study was determined by counting the deaths of children born in the previous 5 years. Therefore, the percentage of subjects reported as deceased was lower than the general reported mortality rate among children under 5 years of age in this county. Potential regional variations in child mortality within the country may also explain the lower than expected mortality rate in the present study.

Our results suggest a lack of maternal autonomy relates to chronic nutritional problems as reflected in its negative associations with linear growth retardation and under-weight. For acute nutritional problems, which are reflected in the indicator for emaciation, the general situation was rather stable. This implied that there were few acutely emerged threats to child nutrition at the time when this study was conducted. Reporting of the occurrence of major illnesses, diarrhoea and respiratory disease, and linear growth retardation generally corresponded to those of other reports from South Asian countries [19,34].

Social customs within families and communities characterise the roles of individuals in the families. As in other countries, the primary caregivers of small children in Afghanistan are their mothers; however, in this country, mothers are subject to a number of restrictions in the decision-making process regarding child healthcare. Women's autonomy has been addressed and its associations with general healthcare-seeking behaviours have been discussed [35]. There is a need for indicators to measure individual practices that reflect multifaceted aspects of autonomy [36]. The results of the present study indicated that particular attention should be paid to maternal autonomy at the household level for the promotion of the health status of children in Afghanistan.

In Afghanistan, marriages form new alliances between families and villages and reinforce social ties to stabilise communities. On the other hand, the pressure perceived by newly married girls and women to bear a child has been widely reported in communities: 18.3% of the mothers included in the present study delivered their first baby before the age of 16, indicating the prevalence of both child marriage and childbearing in childhood in this country. Considerations of human rights issues are relevant in cases of child marriage [37]. Not only the married child but also the children of child-mothers are subject to a number of disadvantages. The poor economic and educational status of these women, and their overall immaturity caused by a lack of learning opportunities may have resulted in difficulties in preventing illness in their children. Culturally appropriate programmes with multifaceted approaches that provide families and communities with education and reproductive health services can help stop child marriage [37], and governments are also expected to play a role in this endeavour.

A physician's gender matters because some religious tenets in Afghanistan restrict contact between men and women who do not have family relationships. It was reported that there were no differences in religious commitment between women who did and did not express a preference for female physicians [38]. Although the advantages of communication between female physicians and female patients in a primary care setting were discussed previously [39], the present study revealed no associations between preference for female physicians and child health. Capacity planning to ensure a balanced distribution of female physicians in local communities is reasonable; however, policies focusing solely on increasing the number of female physicians will not be effective without the means to improve the comprehensive conditions that affect health.

Human security shifts our focus from traditional territorial security to that of the individual. Shortages of food, water and shelter showed a significant association with major illnesses and child growth indicators. Policies to encourage freedom from want and freedom from fear will facilitate the healthy development of children. All people have an inalienable right to sufficient food, adequate shelter, clean water, good health, education for their children, protection against violence and to live without oppression [22]. A lack of basic material needs has been experienced in Afghanistan with devastating effects because of armed conflicts and drought [23], and the long-lasting negative effect of armed conflicts on the health of children should not be neglected.

During the conflicts in Afghanistan, more than 22.7% of the population migrated out of the country, mostly to the neighbouring countries of Pakistan and Iran [8]. Despite the hardships they experienced, the children of those families that were displaced outside the country consistently showed better health than those that were internally displaced. The international community issued a warning against the adverse conditions experienced by internally displaced families [40], although little help reached those in need. Further investigations are required to determine the generality of this result; the findings presented here should be noted to protect the health of ordinary people from the influence of conflicts occurring across the country.

Rural parts of Afghanistan are expected to have further limitations on the mobility of women, attitudes towards maternal autonomy and female health workers [41,42]. An effective healthcare system in this country is urgently required [28]. However, it will not be sufficient to rely merely on building health facilities. Changes are required in the behaviour of men toward female members of their families and within the community. Community-based approaches with a firm understanding of the comprehensive determinants of child health are required. Current country-wide efforts to ensure that all women have access to formal education, the elimination of poverty and the improvement of sanitary conditions should be further enforced.

## Conclusion

The results of the present study in a representative population in Afghanistan revealed a high prevalence of diarrhoea and ARI, emaciation and linear growth retardation among children less than 5 years of age. Family behaviours related to women with regard to child care and household experience of hardships related to morbidity of major illness and the nutritional status of children. A lack of education in mothers, child marriage, lack of maternal autonomy, shortage of basic material needs and internal displacement showed independent significant negative associations with child health variables. Comprehensive efforts centred on the enforcement of legislation to remove the barriers preventing women obtaining healthcare for their children and the satisfaction of basic material needs should be strengthened.

## Abbreviations

ARI: acute respiratory infection; CI: confidence interval; NCHS: National Center for Health Statistics; OR: odds-ratio; SD: standard deviation; WHO: World Health Organization

## Competing interests

The authors declare that they have no competing interests.

## Authors' contributions

TM designed the study, analysed the data, and drafted and revised the manuscript. TT supervised the data analysis and writing. KN participated in data analysis and structured and edited the manuscript. MK prepared the database and participated in the data analysis. SH, MW and KS took part in the preparation of the database.

## Pre-publication history

The pre-publication history for this paper can be accessed here:


